# Herpes zoster as risk factor for dementia: a matched cohort study over 20 years in a 10-million population in Italy

**DOI:** 10.1016/j.tjpad.2025.100167

**Published:** 2025-04-12

**Authors:** Lorenzo Blandi, Paola Bertuccio, Carlo Signorelli, Helmut Brand, Timo Clemens, Cristina Renzi, Anna Odone

**Affiliations:** a School of Public Health, Vita-Salute San Raffaele University, Milan, Italy; b Department of International Health, CAPHRI Public Health and Primary Care Institute, Maastricht University, Maastricht Netherlands; c School of Public Health, Department of Public Health, Experimental and Forensic Medicine, University of Pavia, Pavia, Italy; d Medical Direction, Fondazione IRCCS Policlinico San Matteo, Pavia, Italy

**Keywords:** Dementia, Herpes zoster, Epidemiology, Vaccination, Public health

## Abstract

**Background:**

Herpes Zoster is caused by the reactivation of the Varicella-Zoster Virus. Zoster may influence the occurrence of dementia, but contradictory results about this association emerged from recent studies. These findings did not consider the severity of Zoster and observed individuals for limited follow-up time. Our study used a region-wide Italian registry to investigate the association between severe Zoster infection and dementia occurrence over a 23-year period.

**Methods:**

We included people aged ≥ 50 and hospitalised with Zoster, and two comparison cohorts from both the general population and the hospitalised population without Zoster. By random sampling, the matching 1:5 was based on sex, birth year, and entry date in the cohort. Dementia and Zoster were identified through validated algorithms. A Fine-Gray sub-distribution hazard model was used, accounting for competing risk of death.

**Results:**

We identified 132,968 individuals, of whom 12,088 with severe Zoster, 60,440 matched controls among the general population, and 60,440 matched controls among the hospitalised population. In severe cases of Herpes Zoster, the overall adjusted sub-distributed hazard ratio of dementia was 1.13 (95 % CI 1.07–1.19) compared to the general population, and 1.08 (95 % CI 1.03–1.14) compared to hospitalised population. Hazard ratios were still significant in different strata group, including by sex, age group (including in 50–65 younger adults) and at different follow-up period.

**Conclusions:**

Our population-based study found an increased risk of developing dementia among severe Zoster cases. Those results support the importance of improving Zoster prevention and extending the vaccination recommendations to younger age groups.

## 1 Introduction

Herpes Zoster (HZ) is a disease caused by the reactivation of the Varicella-Zoster Virus (VZV), a neurotrophic virus which latently resides in sensory ganglia after an episode of chickenpox [1]. From previous studies it emerged that the infection of different neurotropic viruses could increase the risk of dementia, especially in hospitalised subjects and within the first years after exposure [[2], [3], [4]]. Therefore, recent studies have also investigated the association between HZ and dementia [5,6], with inconclusive results. In fact, the latest systematic reviews and meta-analyses on the relationship between HZ and dementia reported contradictory results, finding limited association at specific follow-up time or any association at all [[7], [8], [9]]. These reviews included mainly population-based studies, comparing people with HZ to the general population. HZ cases were identified from administrative data by a validated algorithm of ICD-9-CM codes with positive predictive value of 86 % in patients aged 50 and older [10]. However, these reviews reported major limitations, including the small number of studies retrieved and failure to consider the severity of HZ, and a limited follow-up time, highlighting the need for further epidemiological research [7,8].

Understanding dementia risk factors has important public health implications. Few risk factors for dementia have been identified, accounting for just 45 % of the 13 million dementia attributable cases in Europe among older people [11,12]. The incidence rate of HZ (per 1000 person-years) in the general population in Europe is between 7 and 8 in people over 50 years old and notably increase depending on age or comorbidities [13]. Therefore, understanding the association between HZ and dementia is important since a vaccine is available. Nowadays, the HZ vaccination is currently mostly recommended in the general population aged 60–65 or over and in high-risk populations by only few European countries, and its uptake is low or unknown [14,15].

In this context, we aimed to evaluate the association between severe Herpes Zoster infection and dementia occurrence in adults aged 50 or older using population-level data over a 23-year period.

## 2 Methods

### 2.1 Study design

We created a matched cohort study based on administrative health records from the Lombardy Region Datawarehouse. This longitudinal study was performed on an open cohort of individuals aged 50 years or older who were residents in the Lombardy Region between January 1, 2001 and December 31, 2023.

### 2.2 Data source

The Lombardy Region Welfare Directorate collects health records in its comprehensive healthcare system's Datawarehouse from various sources, including hospital discharge registry, drug prescriptions, and other databases [16]. This ensures that all citizens’ interactions with the healthcare system are comprehensively captured. Each record has a unique anonymous alphanumeric ID code linked to every individual. By the matching of the ID codes between different datasets, it is possible to perform record linkage of any information flows and retrieve all data of any individual over the period 2001–2023, in compliance with the General Data Protection Regulation.

### 2.3 Study population, study follow-up

The study population included a total of 7030,374 individuals aged 50 years or older who were residents in the Lombardy Region between January 1, 2001 and December 31, 2023. Individuals entered the open cohort on January 1, 2001 or upon reaching their 50th birthday in the study period (i.e., individuals born in 1973 or earlier).

### 2.4 Variables of interest

The exposure of interest was hospitalisation with a diagnosis of HZ, identified using ICD-9-CM codes 053.2, 053.20, 053.21, 053.22, or 053.29, according to a validated algorithm [10].

The outcome of interest was the incidence of dementia, identified by a validated algorithm based on administrative health data and published by the Italian National Health Institute (see Supplementary Material, Figure S1) [17], [18]. The algorithm integrates information from four different administrative data flows: (i) the Hospital Discharges Registry; (ii) Drugs Prescriptions; (iii) Administrative Exemptions (administrative certificates for selected medical conditions that exempt from payment for health services); and (iv) Long-Term Care Facilities Admissions.

As covariates, we included history at baseline of the main condition and diseases identified as risk factors of dementia or involved in its causal pathway [11]: arrhythmic myocardiopathy, cancer, chronic obstructive pulmonary disease (COPD), depression, diabetes, epilepsy, hearing loss, high-LDL cholesterol, hyper- and hypoparathyroidism, hypertension, immunocompromised status, ischemic heart disease, rheumatoid arthritis, stroke, systemic lupus erythematosus, traumatic brain injury and visual loss. Most of the covariates’ algorithms were retrieved from *Open Data* (see Supplementary Material, Table S1), the official portal of Lombardy to access public data [19].

For exposed individuals, the entry date was defined as the date of their first hospital admission with an HZ diagnosis during the study period. They were followed up until the first occurrence of the outcome (dementia), death, emigration from the Lombardy Region, or the end of the study period (December 31, 2023), whichever came first. Individuals who had a diagnosis of dementia or died prior to the entry date were excluded.

### 2.5 Matching method

A 1:5 matching approach was used based on sex, birth year, and exposed entry date, and a simple random sampling. Exposed subjects were included in Group 1. Two groups of unexposed matched controls were used: i) individuals in the general population, named as Group 2; ii) individuals hospitalized with a diagnosis other than HZ infection, named as Group 3. Each individual from Group 1 was individually matched to five individuals from Group 2, and matched to five individuals from Group 3.

### 2.6 Statistical analysis

Descriptive statistics were computed to summarize baseline characteristics across groups. To estimate the risk of dementia associated with the exposure, we applied Fine-Gray sub-distribution hazard models, accounting for the competing risk of death. These models were stratified by the matching variables (sex, birth year, and entry date) and adjusted for selected potential confounders, including history of arrhythmic cardiomyopathy, cancer, chronic obstructive pulmonary disease (COPD), depression, diabetes, epilepsy, hearing loss, high low-density lipoprotein (LDL) cholesterol, hyper- and hypoparathyroidism, hypertension, immunocompromised status, ischemic heart disease, rheumatoid arthritis, stroke, systemic lupus erythematosus, traumatic brain injury, and visual impairment. The results were reported as sub-distribution hazard ratios (SHRs) with 95 % confidence intervals (CIs). Given that the proportional hazards assumption was not satisfied over the entire follow-up period, we estimated SHRs separately at different time intervals. Specifically, SHRs were estimated for the overall follow-up period, and within the following time intervals: 0–1 year, 0–10 years, 1–10 years, and 10–23 years. To assess the cumulative incidence of dementia while accounting for death as a competing event, we computed cumulative incidence functions (CIFs) stratified by groups over the entire follow-up period. Additionally, CIFs were estimated at 1 year and 10 years, considering the occurrence of dementia or death within these timeframes while censoring individuals without events at the end of each period. Stratified analyses were conducted by sex, and two age groups (50–65 and 66+ years). Fine-Gray models were performed separately to compare Group 1 vs. Group 2, and Group 1 vs. Group 3. All the analyses were performed using the statistical software SAS 9.4.

## 3 Results

We identified 132,968 individuals, of whom 12,088 in Group 1, 60,440 matched controls among Group 2, and 60,440 matched controls among Group 3. The mean age was 74.6 years (total range 50–104 years) and 57.6 % were women (see Table 1). At baseline, cancer, COPD, depression, immunocompromised status and rheumatologic diseases were more prevalent in Group 1 than in Group 2 and 3.

**Table 1:** Selected baseline characteristics of people diagnosed with HZ and two matched comparison cohort, Lombardy, Italy 2001–2023.

	Hospitalised with HZ	General Population	Hospitalised without HZ
**Groups**	1	2	3
Exposure to HZ	Exposed	Unexposed	Unexposed
**Counts, n**	12,088	60,440	60,440
**Sex, n (%)**			
Male	5125 (42.4)	25,625 (42.4)	25,625 (42.4)
Female	6963 (57.6)	34,815 (57.6)	34,815 (57.6)
**Age at baseline, years**			
Mean (SD)	74.6 (10.4)	74.6 (10.4)	74.6 (10.4)
Age range	50–104	50–104	50–104
**Age groups, n (%)**			
50–65	12,705 (21.0)	2541 (21.0)	12,705 (21.0)
66–80	28,505 (47.2)	5701 (47.2)	28,505 (47.2)
81+	19,230 (31.8)	3846 (31.8)	19,230 (31.8)
**Follow-up time, person-months**			
Mean (SD)	80.6 (72.3)	75.6 (70.5)	103.7 (73.3)
Overall person-months	913,604	6268,891	4874,729
**Comorbidities prevalence at baseline, n (%)**			
Arrhythmic myocardiopathy	3042 (25.2)	8679 (14.4)	15,174 (25.1)
Cancer	3795 (31.4)	8777 (14.5)	16,808 (27.8)
COPD	6468 (53.5)	24,410 (40.4)	29,587 (48.9)
Depression	4171 (34.5)	14,026 (23.2)	17,965 (29.7)
Diabetes	3017 (25.0)	10,018 (16.6)	13,900 (23.0)
Epilepsy	394 (3.3)	660 (1.1)	1262 (2.1)
Hearing loss	0 (0)	2 (0)	6 (0.0)
High LDL cholesterol	4555 (37.7)	19,596 (32.4)	23,452 (38.8)
Hyper and Hypoparathyroidism	38 (0.3)	82 (0.1)	171 (0.3)
Hypertension	9921 (82.1)	43,198 (71.5)	48,826 (80.8)
Immunocompromised status	1491 (12.3)	1069 (1.8)	3398 (5.6)
Ischemic heart disease	3502 (29.0)	10,639 (17.6)	17,369 (28.7)
Rheumatoid arthritis	344 (2.8)	460 (0.8)	920 (1.5)
Stroke	716 (5.9)	1568 (2.6)	3050 (5.0)
Systemic lupus erythematosus	49 (0.4)	53 (0.1)	86 (0.1)
Traumatic brain injury	255 (2.1)	689 (1.1)	1137 (1.9)
Visual loss	7 (0.1)	20 (0.0)	27 (0.0)

Over the study period, a total of 1606 new cases of dementia and 6772 deaths occurred among Group 1, 7636 new cases of dementia and 23,505 deaths among Group 2, and 7481 new cases of dementia and 31,775 deaths among Group 3 (see supplementary material, Table S2).

**Fig. 1:**
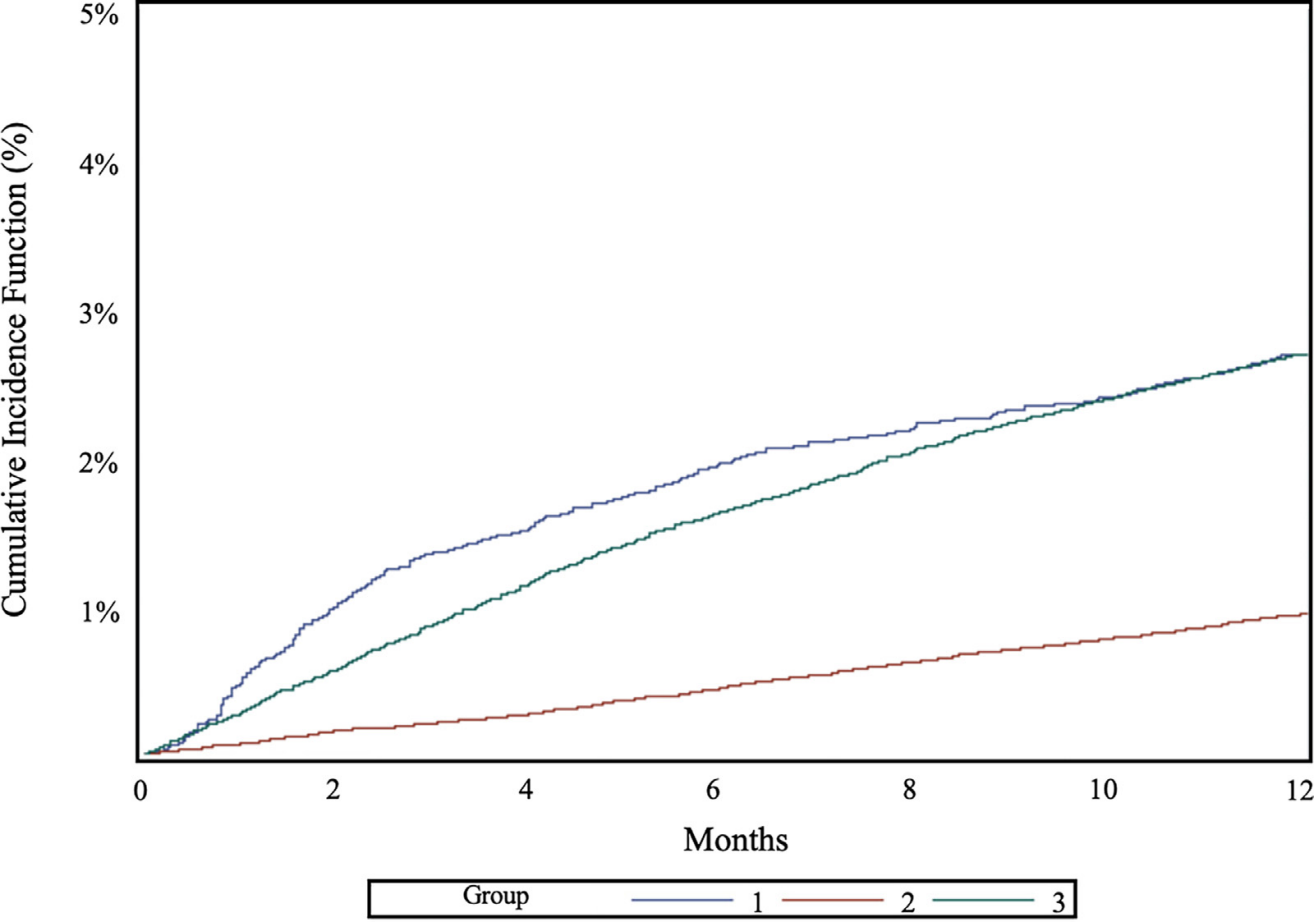
Cumulative Incidence Curves for People Diagnosed with HZ (Group 1) and two matched controls groups (Group 2 and Group 3), within the *0–1 years follow-up* subpopulation, in Lombardy, Italy 2001–2023, accounting for Death as a Competing Risk.

**Fig. 2:**
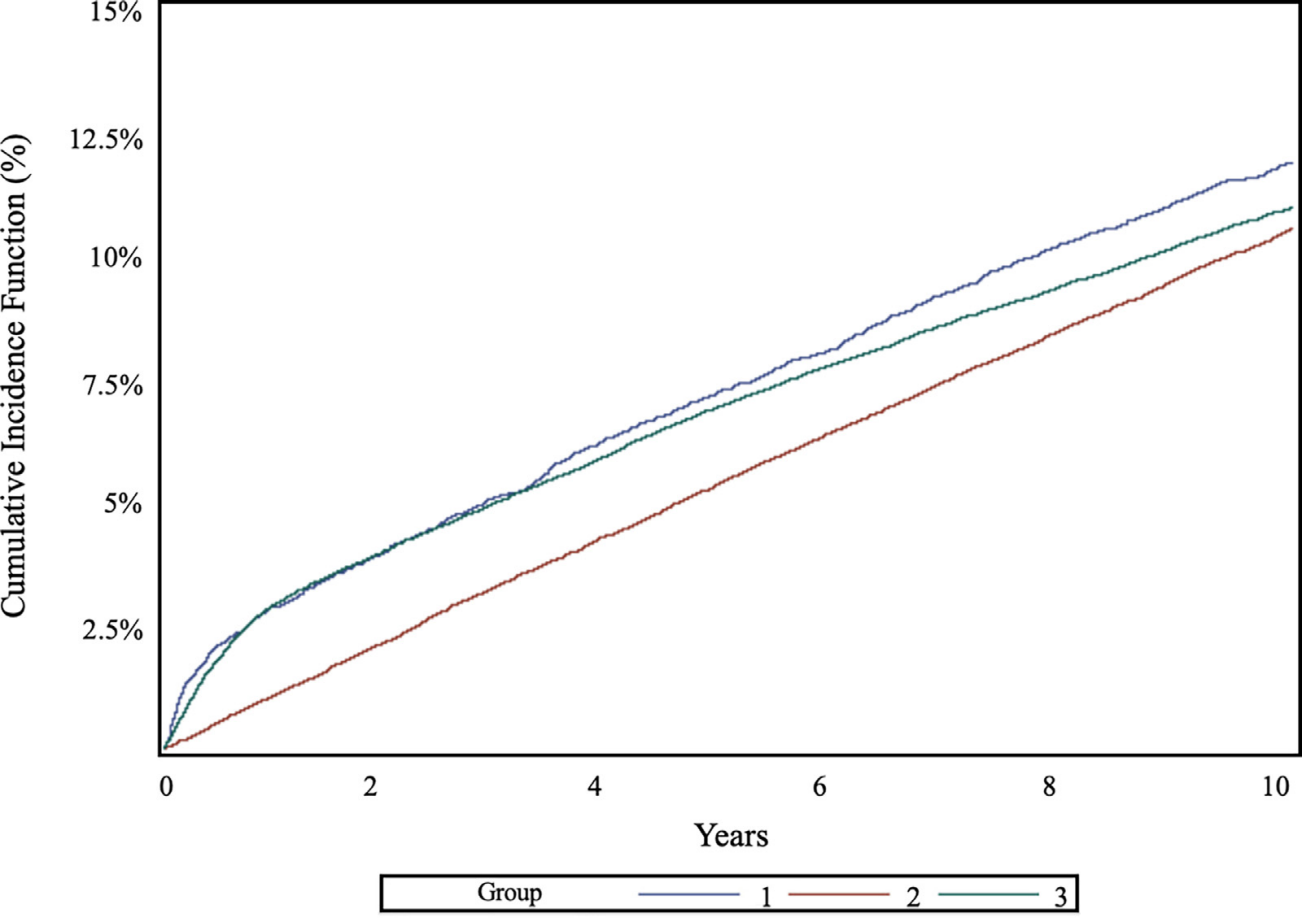
Cumulative Incidence Curves for People Diagnosed with HZ (Group 1) and two matched controls groups (Group 2 and Group 3), within the *0–10 years follow-up* subpopulation, in Lombardy, Italy 2001–2023, accounting for Death as a Competing Risk.

The overall cumulative incidence function of dementia was 17.9 % (95 % CI 17.0–18.8) among Group 1, 19.3 % (95 % CI 18.8–19.7) among Group 2, and 17.0 % (95 % CI 16.6–17.5) among Group 3 (see Supplementary Material, Figure S2). The 1-year cumulative incidence function of dementia was 2.9 % (95 % CI 2.6–3.2) among Group 1, 1.1 % (95 % CI 1.0–1.2) among Group 2, and 3.0 (95 % CI 2.8–3.1) among Group 3 (see Fig. 1). The 10-years cumulative incidence function of dementia was 11.9 % (95 % CI 11.3–12.6) among Group 1, 10.6 % (95 % CI 10.3–10.8) among Group 2, and 11.0 % (95 % CI 10.7–11.3) among Group 3 (see Fig. 2).

Adjusted SHRs for dementia associated with HZ (Group1) as compared to Group 2 and Group 3, over the whole follow-up period, and at different time intervals, along with stratified estimates, are presented in Table 2. No substantial differences between estimates from unadjusted and adjusted models emerged (see supplementary material, Table S3). The overall adjusted SHR of dementia was 1.13 (95 % CI 1.07–1.19) in subjects hospitalized for HZ (i.e. Group 1), as compared to Group 2, and 1.08 (95 % CI 1.03–1.14) as compared to Group3. Stratified SHRs by sex and age groups showed consistent results.

**Table 2:** Sub-distribution Hazard Ratios (HR) of dementia incidence and corresponding 95 % confidence intervals (CI) associated with Herpes Zoster (Group 1), as compared to Group 2 and Group 3, with stratified estimates. Lombardy, Italy. 2001–2023.

	Group 1 vs. Group 2		Group 1 vs. Group 3	
	SHR (95 % CI)	*P* value	SHR (95 % CI)	*P* value
**Overall FU period**	**1.13 (1.07–1.19)**	**<0.001**	**1.08 (1.03–1.14)**	**0.001**
Males	1.14 (1.04–1.25)	0.006	1.12 (1.03–1.22)	0.012
Females	1.13 (1.06–1.20)	<0.001	1.07 (1.01–1.14)	0.030
50–65 years old	1.23 (0.99–1.52)	0.061	1.22 (1.01–1.47)	0.045
>65 years old	1.12 (1.06–1.18)	<0.001	1.08 (1.02–1.13)	0.006
**0–10 FU years**	**1.22 (1.15–1.29)**	**<0.001**	**1.08 (1.02–1.14)**	**0.007**
Males	1.25 (1.12–1.38)	<0.001	1.13 (1.03–1.25)	0.014
Females	1.21 (1.12–1.30)	<0.001	1.06 (0.99–1.13)	0.103
50–65 years old	1.76 (1.24–2.51)	0.002	1.38 (1.02–1.86)	0.037
>65 years old	1.20 (1.13–1.28)	<0.001	1.07 (1.01–1.13)	0.017
**Other time intervals**	**2.43 (2.15–2.75)**	**<0.001**	**0.97 (0.87–1.08)**	**0.562**
0–1 FU year	2.43 (2.15–2.75)	<0.001	0.97 (0.87–1.08)	0.562
1–10 FU years	1.16 (1.09–1.24)	<0.001	1.11 (1.04–1.19)	<0.01
10–23 FU years	1.27 (1.13–1.43)	<0.001	1.24 (1.10–1.39)	<0.001

Early SHR for dementia, i.e. after 1 year of follow-up, was 2.43 (95 % CI 2.15–2.75) in Group 1 compared to Group 2, and 0.97 (95 % CI 0.87–1.08) in Group 1 compared to Group 3. Stratified analyses showed consistent results, except the strata of people aged 50–65 years, which showed a higher association with a SHR of 7.54 (95 % CI 2.35–24.12). When considering the estimates after 10 years of follow-up, SHRs of dementia were 1.22 (95 % CI 1.15–1.29) in Group 1 compared to Group 2, and 1.08 (95 % CI 1.02–1.14) in Group 1 compared to Group 3. No substantial differences emerged across strata groups.

Between 1 and 10 years of follow-up, the adjusted SHR of dementia was 1.16 (95 % CI 1.09–1.24) in Group 1 compared to Group 2, and 1.11 (95 % CI 1.04–1.19) in Group 1 compared to Group 3. Between 10 years and the end of follow-up (23 years), the adjusted SHR of dementia was 1.27 (95 % CI 1.13–1.43) in Group 1 compared to Group 2, and 1.24 (95 % CI 1.10–1.39) in Group 1 compared to Group 3.

## 4 Discussion

According to our hypothesis, we observed an overall increased risk of dementia among people hospitalised with HZ followed for up to 23 years. In particular, the risk increased of 13 %, as compared to the general population, and of 8 %, as compared with subjects hospitalized for other reason other than HZ. We found a higher risk over all the different study periods in different age groups.

The most recent systematic reviews with meta-analysis performed on observational studies reported both no association and an increased risk of dementia among people with HZ, but the findings from their studies should be dealt with some caution [[7], [8], [9]]. Firstly, different results emerged in subgroup analysis, depending on the antiviral treatments received and on the kind of dementia occurred [9]. Then, among the articles which reported no association, the study populations included people diagnosed at the primary care or outpatient setting [6,20], evaluating different cohorts compared to our study. From a Danish nationwide registry, *Schmidt* et al. found that people with HZ have no association or a slightly reduced risk of dementia compared to people in the general population without HZ, reporting a hazard ratio of 0.98 (95 % CI 0.92–1.04) in the 0–1 years period of follow-up, and of 0.93 (95 % CI 0.90–0.95) in the 1–21 years period. However, these results could be explained by missed HZ's and dementia's diagnoses at the primary care level, as discussed in the limitation of the study [6]. In addition, the sampling technique of the study population could have been affected by selection bias, identifying only two cohort, one exposed and one unexposed, without any inclusion criteria related to clinical severity of HZ. On the other hand, they found that patients exposed to HZ with central nervous system involvement had an almost two-fold increased relative risk of dementia, suggesting that in the most severe cases the association would emerge [6]. *Warren-Gash* et al. found no evidence that HZ was associated with an increased risk of dementia incidence in a large UK population-based matched cohort [20]. In this study, authors evaluated the risk of dementia using a Cox proportional hazards regression model, which fits for low competing risk of death [20]. However, the competing risk of death is high in a frail and aged population with HZ. Other population-based studies, using administrative data from Taiwan, South Korea and US, found that HZ increases the risk of dementia incidence, consistently with our results [[21], [22], [23]]. Our exposed study population included only those hospitalised with HZ, identifying the most severe cases. That allowed us to compare different kind of populations, including the general population and the more frail and homogenous population of those hospitalised without HZ. In addition, we implemented a Fine-Gray model to consider the high competing risk of death. The use of validated algorithm for case and outcome definition also strengthened the methodology [10,17]. Our study confirmed an overall increased risk of dementia incidence in people exposed to HZ, as other studies previously did [21,23]. A severe case of HZ acted as a risk factor for dementia, both compared to the general population and to hospitalised individuals. Indeed, over the 0–10 and 0–23 years study period, the risk of dementia incidence was greater among exposed individuals to HZ. By observing the different study periods, we took into account for potential biases identified in previous studies, and we focused over the 0–1-, 0–10- and 1–10 years periods. Indeed, comparing these periods, we assessed the effect of first year of follow-up, so evaluating a potential detection bias, and the last 10–23 years, where health profile of individuals could change. Indeed, we found a higher risk of developing dementia within 1 year after hospitalisation with HZ in comparison with the general population. On the other hand, compared to people hospitalised without HZ, we did not find any association. These results support the hypothesis that medical surveillance after hospitalisation might increase the diagnosis of new dementia cases among people hospitalised with or without HZ, resulting in a detection bias. To limit this bias, according to the approach implemented in a similar study [6], we estimated the risk of dementia incidence both over the 0–10 and 1–10 follow-up period. Focusing on the 1–10 follow-up period, computed to exclude the first year of follow-up and to limit the potential detection bias, the risk among exposed was still greater than in the two control groups, confirming a significant impact of HZ.

Currently, several hypotheses exist on the pathophysiological mechanisms of HZ infection that lead to dementia. The reactivation of the virus may induce neuroinflammation, leading to the formation of misfolded oligomers and the accumulation of amyloid plaques and neurofibrillary tangles [24]. VZV may directly infect astrocytes, facilitating the generation of internal amyloid and the aggregation of amyloid fibrils in the extracellular environment [25]. A different hypothesis suggests that herpes zoster, particularly when it affects cranial nerves, induces cerebral vasculopathy and stroke, leading to neurological damage [22,26]. Other studies hypothesised an increased production of amyloid plaques either directly or through binding to insulin-degrading enzyme [21,22], or induction of systemic inflammatory cytokines [22,27]. Severe herpes zoster affecting the central nervous system may lead to encephalopathy and consequently to dementia, similarly to HIV or syphilis [28]. *Schmidt* et al. investigated these pathways, suggesting that neuroinflammation or microinfarction (without clinical symptoms) could play as mediator between HZ affecting the central nervous system and dementia, instead of a vasculopathy mediated by stroke or other cerebrovascular major episodes [6]. Moreover, they identified a higher risk within the first year of follow-up after the exposure, however a detection bias resulting from intensive clinical examination in the acute phase may all be responsible for the reported particularly strong association [6]. Our study observed a similar strong association in the first 1 and 10 years of follow-up. In addition, we adjusted our model for stroke and other vascular diseases, and we limited the diagnosis bias by comparing our exposed cases to hospitalized controls without HZ. Considering the timing of dementia incidence, our study suggested that the reactivation of the virus may induce neuroinflammation and its acute and subacute effect lead to dementia. However, our hypothesis is limited and similar to studies based on administrative data or which reported an association only within the first year. The current literature described different biological mechanisms and a recent systematic review underlined that this pathway is still unclear [9], so further epidemiological studies are encouraged.

From a clinical perspective, it is important to contextualize our findings. Indeed, when compared to the general population (group 2), the increased risk of dementia was notably higher among people with severe HZ. However, when compared to the hospitalised population (group 3), they showed a small increase of risk. These findings provide limited suggestions to clinicians. Given the short-term effect of concurrent severe HZ and hospitalisation status, vaccination would be suggested at the time of the patient's discharge from hospital. On the other hand, from a public health perspective, the available evidence underlines the importance of vaccination to prevent HZ. Indeed, this small risk in a large, aged and partially vaccinated population can lead to a considerably burden of disease. To date, the recommendation for such vaccination in Europe is heterogeneous and often targeted at those over 60 or 65 years of age and selected high-risk populations [14]. Our study showed that the increased risk is also in the younger age group. This supports the revision of immunization strategies, because it suggests the extension of vaccination to people between 50 and 65 years of age. To best of our knowledge, our population-based matched cohort study is the first evidence which found a clear association between HZ and dementia incidence in people aged 50–65. These results have important public health implications, also considering that dementia counts 55 million worldwide and the burden of disease is projected to increase threefold by 2050 [29,30]. Further studies should investigate dementia and other diseases causing neuroinflammation to find common pathophysiological mechanisms and confirm our evidence.

Our study has several strengths. We identified all hospitalised patients with HZ over a period of 23 years in the Lombardy region. We conducted a matched-cohort study with 1:5 random sampling, allowing us to assess the association between HZ and dementia in a large cohort. Matching was possible on all necessary variables also identified in other studies, i.e. calendar year, age, sex and period of hospitalisation [20,31]. We adjusted the model for several covariates that are implicated in an increased risk of dementia [11]. We checked for differences between the unadjusted and adjusted model. The data source allowed us to retrieve administrative data on all health services provided for the entire population covered by the Italian National Health Service. Our study has also some limitations, including its retrospective study design and being based on administrative data used for secondary purposes. Administrative data may be influenced by misclassification bias. Misclassification of exposure or outcome can dilute or exaggerate associations, especially if it's differential across groups. To overcome this limitation, we used validated algorithms for both case and outcome definitions [10,17]. Our study could not retrieve data on the medical treatments received for HZ, nor about vaccines administered to prevent HZ. These may have had an impact in reducing the risk of dementia in those exposed to HZ, as reported by other authors [9,21,22]. Our study did not consider also data from outpatient services. This may lead to a selection biases, including only severe cases seeking for acute care. When available, further epidemiological studies should consider information on preventive interventions and medical treatments, and retrieve data from outpatient services. Our study does not examine the effect of other confounders, including socio-economic factors, educational level, lifestyle, and other identified risk factors [11]. A Danish population-based study did not find any association between these factors and HZ, while other studies from UK and Spain reported opposite findings [[32], [33], [34]]. At the same time, dementia is associated with social deprivation, so the risk of dementia in our population could be under- or overestimated [11]. However, we adjusted for many comorbidities which are associated to several social determinants. Adding further social variables in future studies will allow to adjust estimates with a more comprehensive approach. In addition, we considered comorbidities at the individuals’ entry in the open cohort, but we did not include the incidence of these comorbidities over the follow-up period, thus possibly missing any change in the risk profile of individuals. However, our results were stratified by different follow-up periods and showed a significant association between exposure and outcome, adding new evidence to the literature of the HZ impact on dementia incidence.

## 5 Conclusions

Our population-based study shows that severe HZ cases have an increased risk of developing dementia when compared to both the general population and the hospitalised population, with people with HZ aged between 50 and 65 years being at higher risk. Those findings support improving immunization public health strategies and extending the vaccination recommendations to younger people. This risk remains significantly higher over the whole study period, especially during the first 10-years of follow-up.

## CRediT authorship contribution statement

**Lorenzo Blandi:** Writing – review & editing, Writing – original draft, Project administration, Methodology, Investigation, Funding acquisition, Formal analysis, Data curation, Conceptualization. **Paola Bertuccio:** Writing – review & editing, Writing – original draft, Visualization, Validation, Supervision, Software, Methodology, Formal analysis, Data curation, Conceptualization. **Carlo Signorelli:** Writing – review & editing, Validation, Supervision, Resources, Conceptualization. **Helmut Brand:** Writing – review & editing, Validation, Supervision, Conceptualization. **Timo Clemens:** Writing – review & editing, Validation, Supervision, Conceptualization. **Cristina Renzi:** Writing – review & editing, Validation, Supervision, Methodology, Conceptualization. **Anna Odone:** Writing – review & editing, Validation, Supervision, Methodology, Conceptualization.

## Declaration of competing interests

The authors declare the following financial interests/personal relationships which may be considered as potential competing interests: Lorenzo Blandi reports administrative support was provided by Lombardy Region. If there are other authors, they declare that they have no known competing financial interests or personal relationships that could have appeared to influence the work reported in this paper.

## Appendix Supplementary materials

Image, application 1
