# Supplementary material for: Herpes zoster as risk factor for dementia: a matched cohort study over 20 years in a 10-million population in Italy
Source: J Prev Alzheimers Dis. 2025 Apr 12;12(6):100167. doi: 10.1016/j.tjpad.2025.100167 (PMC12434264; doi:10.1016/j.tjpad.2025.100167)
Supplement: Supplementary file 1 [file mmc1.docx]

**SUPPLEMENTARY MATERIALS**


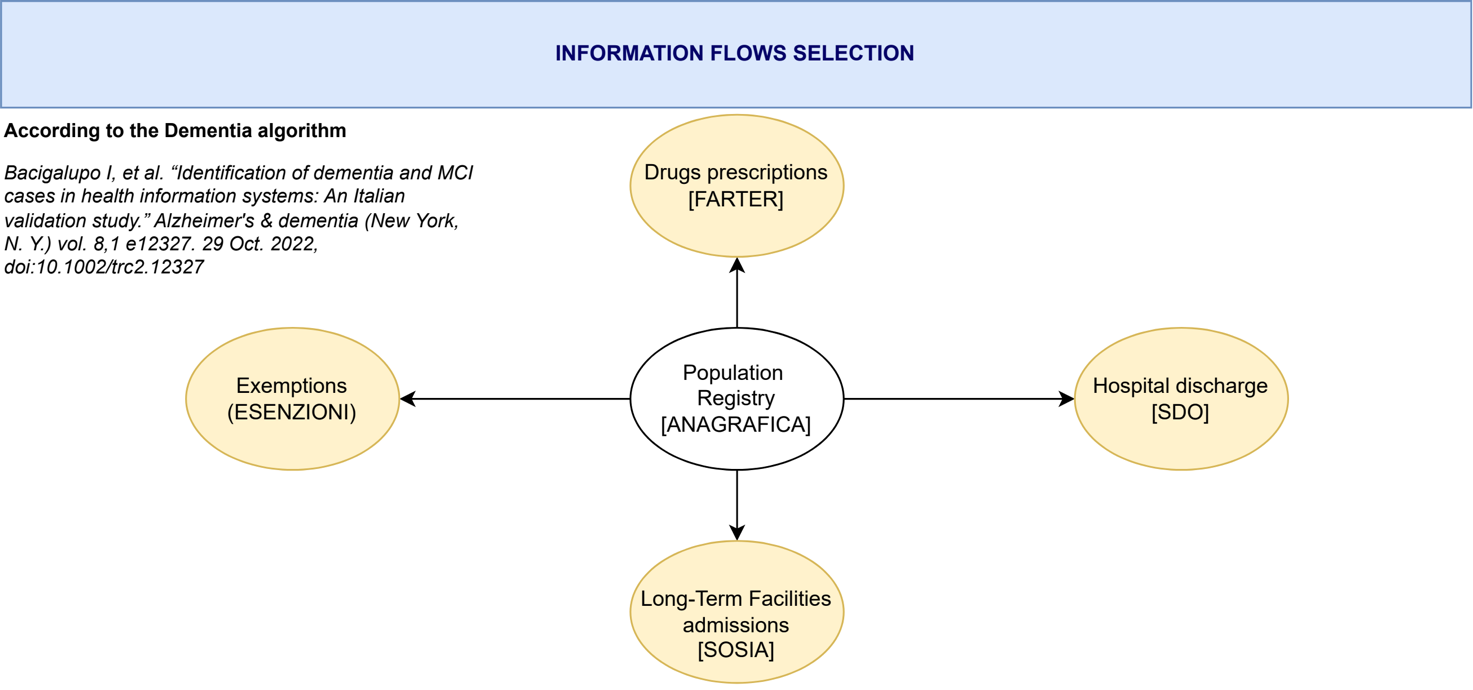


**Figure S1.** Dementia case definition, according to a validated algorithm.

**
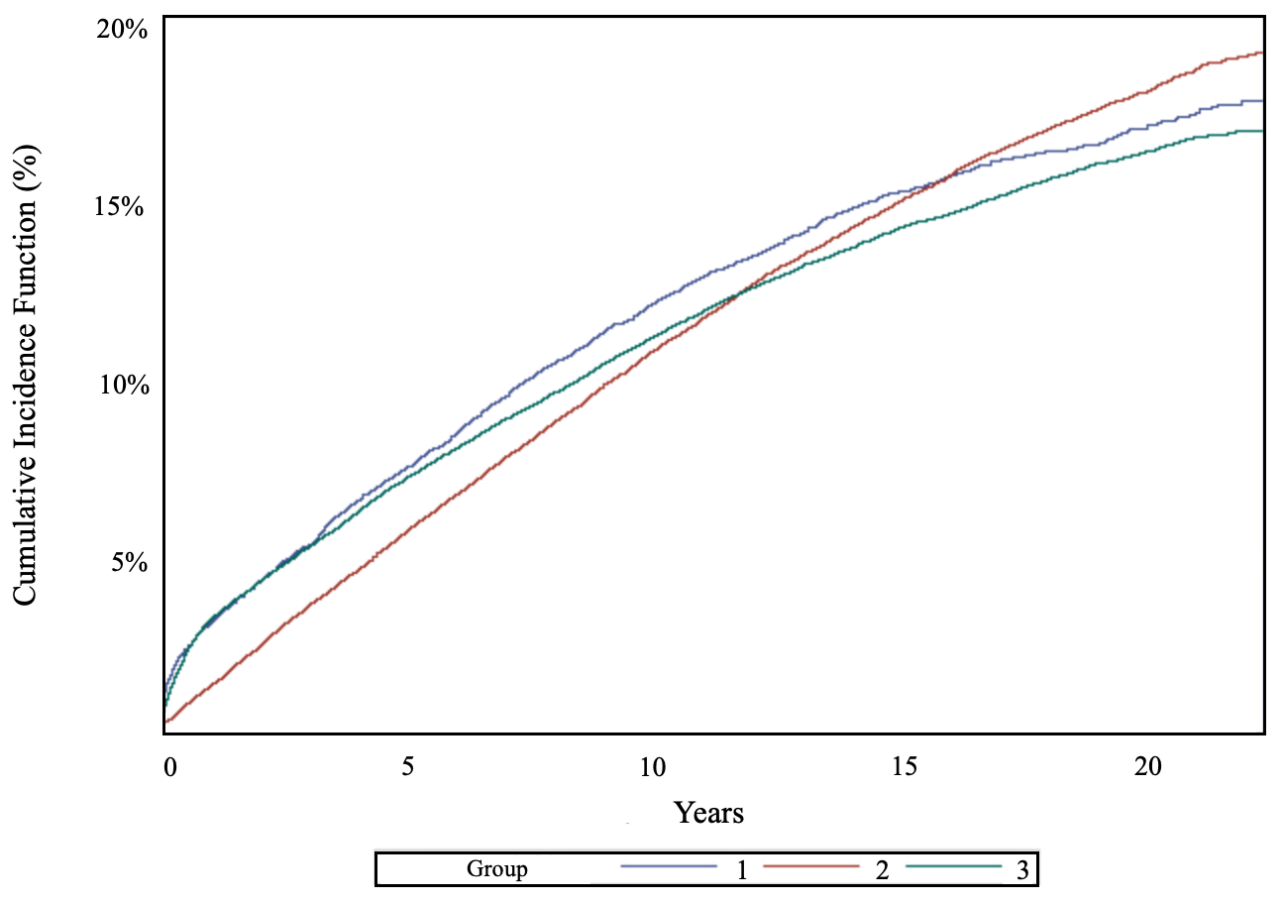
**

**Figure S2.** Overall Cumulative Incidence Curves for People Diagnosed with HZ (Group 1) and two matched controls groups (Group 2 and Group 3), in Lombardy, Italy 2001–2023, accounting for Death as a Competing Risk.

**Table S1.** Technical details and sources to identify covariates

| **Covariate** | **Technical details** | **Sources** |
| --- | --- | --- |
| Arrhythmic myocardiopathy | Algorithm from multiple data sources: Drug Prescriptions, Hospital Discharges Registry, Administrative Exemptions, Outpatient Services | Open Data, Lombardy |
| Cancer | Algorithm from multiple data sources: Drug Prescriptions, Hospital Discharges Registry, Administrative Exemptions, Outpatient Services | Open Data, Lombardy |
| Chronic obstructive pulmonary disease | Algorithm from multiple data sources: Drug Prescriptions, Hospital Discharges Registry, Administrative Exemptions | Open Data, Lombardy |
| Depression | Use of antidepressive drugs from Drug Prescription | Ad-hoc elaboration |
| Diabetes | Algorithm from multiple data sources: Drug Prescriptions, Hospital Discharges Registry, Administrative Exemptions | Open Data, Lombardy |
| Epilepsy | Algorithm from multiple data sources: Drug Prescriptions, Hospital Discharges Registry, Administrative Exemptions | Open Data, Lombardy |
| Hearing loss | Administrative exemption | Ad-hoc elaboration |
| High-LDL cholesterol | Use of cholesterol lowering drugs | Ad-hoc elaboration |
| Hyper- and hypoparathyroidism | Algorithm from multiple data sources: Hospital Discharges Registry, Administrative Exemptions | Open Data, Lombardy |
| Hypertension | Algorithm from multiple data sources: Drug Prescriptions, Hospital Discharges Registry, Administrative Exemptions | Open Data, Lombardy |
| Immunocompromised status | Hospital Discharges Registry | Agency for Healthcare Research and Quality indicator |
| Ischemic heart disease | Algorithm from multiple data sources: Drug Prescriptions, Hospital Discharges Registry, Administrative Exemptions | Open Data, Lombardy |
| Rheumatoid arthritis | Algorithm from multiple data sources: Hospital Discharges Registry, Administrative Exemptions | Open Data, Lombardy |
| Stroke | Hospital Discharges Registry | Open Data, Lombardy |
| Systemic lupus erythematosus | Algorithm from multiple data sources: Hospital Discharges Registry, Administrative Exemptions | Open Data, Lombardy |
| Traumatic brain injury | Hospital Discharges Registry | Open Data, Lombardy |
| Visual loss | Administrative exemption | Ad-hoc elaboration |

**Table S2.** New cases of dementia and deaths in People Diagnosed with HZ (Group 1) as compared to two matched controls groups (Group and Group 3), in Lombardy, Italy 2001–2023.

|  | **New cases of dementia** | **Deaths** | **Censored** | **Overall** |
| --- | --- | --- | --- | --- |
| **Group 1** | 1,606 | 6,772 | 3,710 | 12,088 |
| **Group 2** | 7,636 | 23,505 | 29,299 | 60,440 |
| **Group 3** | 7,481 | 31,755 | 21,204 | 60,440 |

**Table S3.** Unadjusted and adjusted Sub-distribution Hazard Ratio of dementia incidence and corresponding 95% confidence intervals in People Diagnosed with HZ (Group 1) as compared to two matched controls groups (Group and Group 3), in Lombardy, Italy 2001–2023.

|  | **SHR** | **Lower 95% CI** | **Upper 95% CI** |
| --- | --- | --- | --- |
| **Group 1 vs Group 2** | | | |
| Unadjusted | 1.08 | 1.03 | 1.13 |
| Adjusted | 1.13 | 1.07 | 1.19 |
| **Group 1 vs Group 3** | | | |
| Unadjusted | 1.08 | 1.03 | 1.14 |
| Adjusted | 1.08 | 1.03 | 1.14 |
